# EHMTI-0055. Abnormal thalamic function in patients with vestibular migraine

**DOI:** 10.1186/1129-2377-15-S1-E32

**Published:** 2014-09-18

**Authors:** A Russo, V Marcelli, F Esposito, V Corvino, L Marcuccio, A Giannone, R Conforti, E Marciano, T Gioacchino, A Tessitore

**Affiliations:** 1 Department of Medical Surgical Neurological Metabolic and Aging Sciences, Second University of Naples, Naples, Italy; 2 Department of Neuroscience, University of Naples ‘‘Federico II’’, Naples, Italy; 3 Department of Medicine and Surgery, University of Salerno, Baronissi (Salerno), Italy; 4 Department of Clinical and Experimental Medicine and Surgery "F. Magrassi-A. Lanzara", Second University of Naples, Naples, Italy

## Introduction

Vestibular migraine (VM) has been increasingly recognized as a possible cause of episodic vertigo, but its pathophysiology is still unclear.

## Aims

We used advanced non-invasive neuroimaging to examine the functional response of neural pathways associated with vestibular stimulation in patients with VM.

## Methods

Twelve patients with VM underwent whole-brain blood oxygen level-dependent (BOLD) fMRI during ear irrigation with cold water. The functional response of neural pathways to this stimulation in patients with VM was compared to age- and gender-matched patients with migraine without aura (MwoA) and healthy controls (HC). Secondary analyses explored associations between BOLD signal change and clinical features of migraine in patients.

## Results

We observed a robust cortical and subcortical pattern of BOLD signal change in response to ear irrigation across all participants. Patients with VM showed significantly increased thalamic activation in comparison with both patients with MwoA and HC. The magnitude of thalamic activation was positively correlated with the frequency of migraine attacks in patients with VM.

## Conclusions

We provide novel evidence for abnormal thalamic functional response to vestibular stimulation in patients with VM. These functional abnormalities in central vestibular processing may contribute to VM pathophysiology.

No conflict of interest.

